# Force-Control vs. Strength Training: The Effect on Gait Variability in Stroke Survivors

**DOI:** 10.3389/fneur.2021.667340

**Published:** 2021-07-15

**Authors:** Prakruti Patel, Agostina Casamento-Moran, Evangelos A. Christou, Neha Lodha

**Affiliations:** ^1^Department of Health and Exercise Science, Colorado State University, Fort Collins, CO, United States; ^2^Department of Applied Physiology and Kinesiology, University of Florida, Gainesville, FL, United States

**Keywords:** motor training, rehabilitation, intervention, locomotor, paresis, motor control, steadiness, walking

## Abstract

**Purpose:** Increased gait variability in stroke survivors indicates poor dynamic balance and poses a heightened risk of falling. Two primary motor impairments linked with impaired gait are declines in movement precision and strength. The purpose of the study is to determine whether force-control training or strength training is more effective in reducing gait variability in chronic stroke survivors.

**Methods:** Twenty-two chronic stroke survivors were randomized to force-control training or strength training. Participants completed four training sessions over 2 weeks with increasing intensity. The force-control group practiced increasing and decreasing ankle forces while tracking a sinusoid. The strength group practiced fast ankle motor contractions at a percentage of their maximal force. Both forms of training involved unilateral, isometric contraction of the paretic, and non-paretic ankles in plantarflexion and dorsiflexion. Before and after the training, we assessed gait variability as stride length and stride time variability, and gait speed. To determine the task-specific effects of training, we measured strength, accuracy, and steadiness of ankle movements.

**Results:** Stride length variability and stride time variability reduced significantly after force-control training, but not after strength training. Both groups showed modest improvements in gait speed. We found task-specific effects with strength training improving plantarflexion and dorsiflexion strength and force control training improving motor accuracy and steadiness.

**Conclusion:** Force-control training is superior to strength training in reducing gait variability in chronic stroke survivors. Improving ankle force control may be a promising approach to rehabilitate gait variability and improve safe mobility post-stroke.

## Introduction

Steady and consistent gait pattern allows humans to walk safely (1–3). In healthy adults, over-ground walking is characterized by relatively small temporal and spatial variability in consecutive strides (4, 5). However, individuals with gait dysfunction demonstrate increased stride-to-stride fluctuations known as gait variability. Exacerbated gait variability leads to poor dynamic balance, unsteady walking, and heightened risk of falls (5). Falls can cause injuries, impair functional mobility, and increase the fear of future falls (6). Consequently, reducing gait variability constitutes an important target for stroke rehabilitation. Until recently (7), stroke locomotor rehabilitation has primarily targeted improvements in global measures of walking performance such as gait speed and distance rather than gait variability (8, 9). Despite improvements in walking speed, stroke survivors may continue to exhibit significant impairments in step variability. For example, a recent study in stroke survivors reported higher variability in step length and stance time, even with relatively high walking speeds of 0.83–1.25 m/s (10). Similarly, another study reported heightened variability in step length, swing time, and stride time even in independently ambulating stroke survivors (11). Thus, stroke survivors can achieve independent ambulation but continue to show significant gait variability that may predispose them to a greater risk of falls (12). Even though improved gait speed is a desirable rehabilitation outcome post-stroke, identifying interventions that reduce gait variability would be crucial to promote safe walking.

Two primary motor deficits linked with impaired gait are declines in motor control and strength (13–18). Motor control deficits manifest as reduced accuracy and consistency of the paretic limb (19–21). Diminished force accuracy and steadiness of lower extremity was linked to impaired mobility and postural instability in older adults (22, 23). Accordingly, force-control training has shown to improve the motor steadiness of the lower limb (24) and manual function in older adults (25, 26). In stroke survivors, increased force fluctuations of hip muscles were related with reduced gait speed and poor dynamic balance (16). Furthermore, motor accuracy of single-joint ankle movements was associated with over-ground walking in stroke (27). Recent work from our group showed that impaired ankle steadiness, but not ankle strength, contributes to increased stride-length variability during over-ground walking in high-functioning stroke survivors (15). Despite the evidence suggesting functional importance of motor accuracy and consistency after stroke, whether a motor intervention that trains accurate and consistent modulation of lower-limb forces could reduce gait variability is unknown. This question constitutes the logical, next step for the rehabilitation of gait variability and is directly addressed in the current study (14).

In addition to motor control deficits, individuals with stroke demonstrate diminished strength or motor weakness (28). Strength deficits manifest as reduced force generation capacity with the paretic limb and are associated with increased postural sway and slower gait speed (29–31). Mounting evidence suggests that strength training is effective in improving gait speed and distance in stroke survivors (32–34). A meta-analysis reported that ballistic strength training positively influences walking speed after stroke (35). Evidence thus far suggests that strength training improves walking speed post-stroke. However, a relatively less investigated outcome of strength training is its impact on gait variability in stroke survivors. Previously, 6 weeks of strength training of the knee extensors improved motor steadiness in healthy older adults (36). However, it remains unknown if strength training could improve motor steadiness and consequently reduce gait variability in stroke survivors. Thus, we aimed to test strength training as another potential intervention for reducing gait variability following stroke in the current study.

Our study adopts an impairment-based approach to identify interventions that improve gait variability after stroke. Previous studies have established that gait training through task-specific movements, balance retraining, and functional strengthening are effective for improving walking ability after stroke (9, 37, 38). Relative to the widely used gait training approaches, we target two specific but distinct motor impairments that limit walking ability in stroke survivors—strength and motor control (accuracy and steadiness). The force-control training focused on gradually increasing and decreasing forces for accurate and steady modulation of submaximal forces. In contrast, strength training focused on generating maximum force through rapid muscle contractions. Our approach of comparing force-control and strength training will provide insights into the development of interventions that target specific motor impairments for improving gait variability in chronic stroke survivors.

The purpose of this study was to compare the effectiveness of two distinct motor interventions for improving gait variability in stroke. Specifically, we investigate the effect of force-control training and strength training on spatiotemporal measures of gait variability in ambulatory chronic stroke survivors. We hypothesized that both force-control and strength training will improve gait variability; however, the magnitude of improvement will be greater with force-control training. This hypothesis was based on our previous work showing gait variability in stroke survivors is associated with ankle movement steadiness and accuracy, but not ankle strength (15). Furthermore, we expected both groups to demonstrate task-specific gains in strength, ankle movement accuracy, and steadiness. The current study is important because it identifies promising motor interventions to reduce gait variability and promote safe mobility in individuals with stroke.

## Methods

### Participants

Twenty-two chronic stroke survivors volunteered to participate in the study. The number of participants was determined based on the sample size recommendations for pilot studies (39). The inclusion criteria were as follows: (1) onset of stroke ≥6 months before study enrollment, (2) ability to walk with or without an assistive device, (3) presence of a minimum of 10° of ankle plantarflexion–dorsiflexion range, (4) ability to follow a three-step command, and (5) not participating in any other form of physical rehabilitation. Exclusion criteria were presence of (1) uncorrected vision and hearing loss, (2) visual neglect, (3) sensory or global aphasia, and (4) pain or musculoskeletal or any other neurological disorder. Prior to participation, all participants read and signed an informed consent that was approved by the Institutional Review Board of the University of Florida.

### Study Design

**Figure 1:**
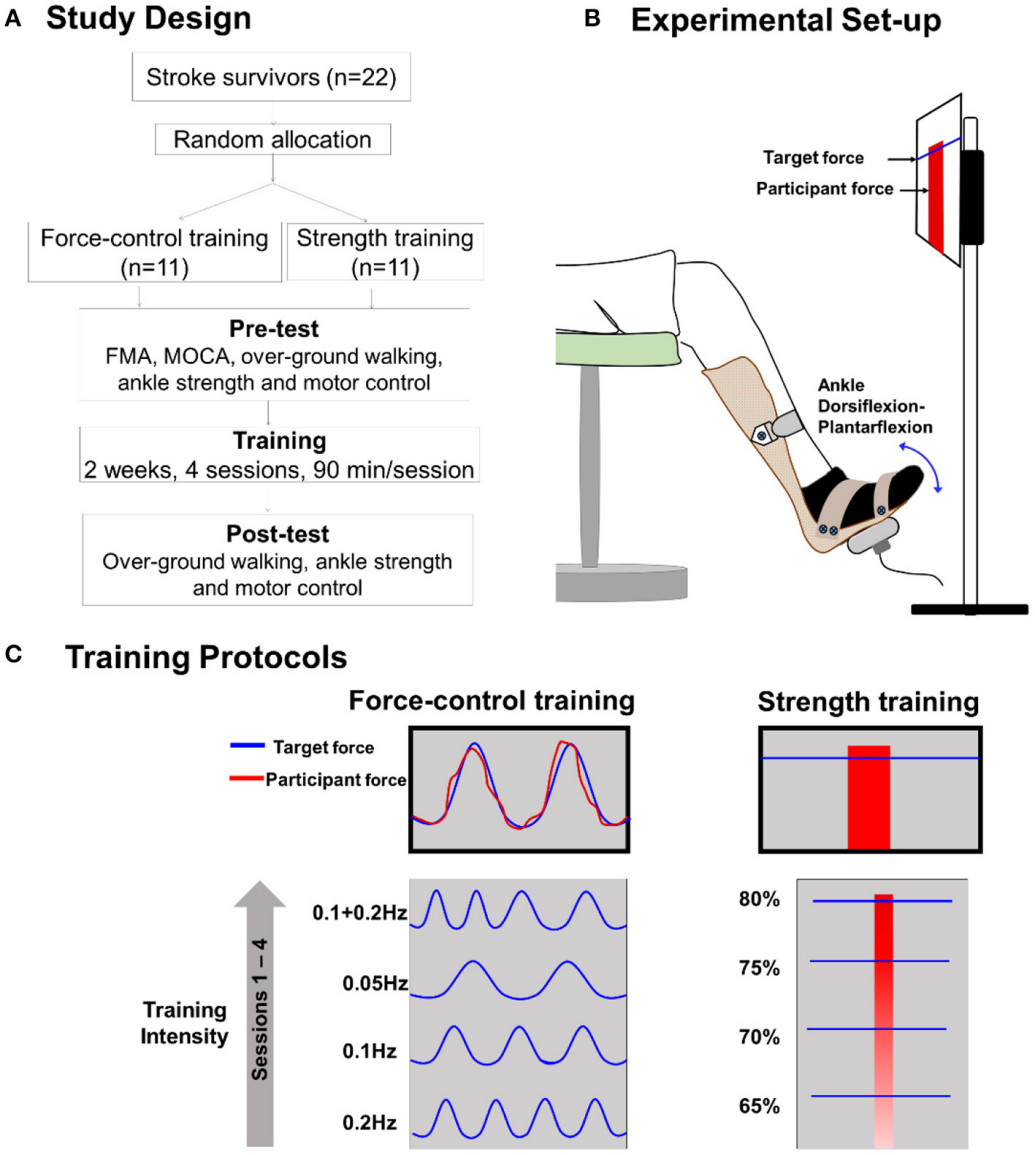
**(A)** Study design. We randomly allocated 22 individuals with stroke to force-control or strength training. **(B)** Experimental setup: participant position while performing isometric ankle plantarflexion and dorsiflexion tasks. The participant received visual feedback on a computer screen that presented the target force and participant forces while performing force-control or strength training tasks. **(C)** Training description: The force-control training group performed a visuomotor force tracking task that involved matching the participant's ankle force to a sinusoidal force trajectory (left). The difficulty of force-control training progressed from a frequency of 0.2, 0.1, 0.05, to 0.1 +0.2 Hz over four sessions. The strength training group performed rapid muscle contractions to reach a target force ranging from 65, 70, 75, to 80% of maximum voluntary contraction (MVC) force over four sessions (right). Participants received visual feedback of their force with a red bar. Both the groups performed the trainings with each leg (paretic and non-paretic) and contraction type (dorsiflexion and plantarflexion).

Figure 1A shows the study design. We assigned the participants to the force-control training or strength training group through simple randomization. Each group received training over 2 weeks in four sessions with increasing intensity. The training duration of each session was 90 min. Participants were evaluated before (pre-training) and after (post-training) the training on over-ground walking, ankle strength, and motor control tasks. Before training, we also performed clinical assessments including the lower extremity subsection of Fugl-Meyer Motor Assessment (FMA-LE) to determine the degree of motor impairments in leg and foot as well as Montreal Cognitive Assessment (MoCA) to determine their global cognitive status.

### Training Description

Figure 1C shows a schematic of the force-control and strength training. The force-control group practiced a visuomotor task that involved tracking a sinusoid. The strength training group practiced fast motor contractions at a percent of participants' maximal voluntary contraction (MVC) force. Both trainings involved unilateral, isometric contraction of the ankle while avoiding any extraneous movements at the knees, hips, and trunk (40). The training protocols involved practicing plantarflexion and dorsiflexion contractions individually with the paretic and non-paretic ankle. The order of the contraction type and leg condition was randomized between participants for both trainings.

During the training session, participants sat in an upright chair in front of a 32-inch monitor placed about 1.5 m away (Figure 1B). The legs were positioned with the hips and knees at ~90 degrees and ankles in a neutral position, i.e., at 90 degrees between the fifth metatarsal and the fibula. Each foot rested on a custom-built foot device and was secured to the device with straps. The plantarflexion and dorsiflexion MVC for each leg was assessed at the beginning of each training session to determine the target forces for force-control and strength training.

### Force-Control Training

Force-control training required gradual increase and decrease in ankle forces to perform a visuomotor sinusoidal tracking task. The computer screen displayed a blue sinusoidal line representing the target trajectory with an amplitude of 10% MVC (range 5–25% MVC) (Figure 1C, left). The real-time feedback of participants' performance was presented with a red line. Within each session, participants performed five sets of six trials for each contraction (plantarflexion and dorsiflexion) and leg condition (paretic and non-paretic). Each training set lasted for about 5 min. Participants received 3 min of rest between subsequent sets that was extended if a participant requested. The training intensity was progressed on successive sessions by reducing the frequency of the sinusoidal target. Reducing the frequency of the sinusoidal target increased the task difficulty as participants were required to exert greater voluntarily control to track forces at the slower rate.

### Strength Training

Strength training involved practicing fast ankle muscle contractions to produce a target force. Participants practiced both plantarflexion and dorsiflexion contractions individually with each leg. On the computer screen, the target force was displayed with a blue horizontal line that represented a pre-determined percentage of their MVC force (Figure 1C, right). Participants performed a ballistic ankle contraction to reach the target line. The real-time feedback of participants' performance was displayed with a vertical red bar. Within each session, participants performed 6 sets of 15 repetitions for each contraction and leg condition. Participants received 3 min of rest between subsequent sets that was extended if a participant requested. The training intensity was progressed on successive sessions by increasing the magnitude of target force from 65 to 80% MVC.

### Pre-post Tests

To examine the effect of the two motor interventions on over-ground walking, we measured gait variability and gait speed. To examine the task-specific effects of force-control and strength trainings, we measured motor accuracy, motor steadiness, and ankle strength for both paretic and non-paretic legs. The pre- and post-training tests were performed by a research personnel who was blinded to the participants' training group assignment.

### Over-ground Walking

Participants walked 7 m at a comfortable and self-selected pace. The spatiotemporal gait characteristics were measured using six wearable sensors (APDM Mobility Lab Inc., Portland, OR, USA) placed on the two wrists, two ankles, the sternum, and the lumbar spine (41). Three walking trials were performed with a 90-s rest period between the trials.

### Gait Data Analyses

The number of gait cycles for each trial ranged from 10 to 30 based on the walking speed of the participant. Position and acceleration data from the movement monitors were used to detect gait events. The data were validated at the end of each trial and stored for offline analysis.

### Gait Variability

We measured the spatial gait variability as the coefficient of variation (CV) of stride length of the paretic leg, non-paretic leg, and mean stride length of both legs combined. We measured the temporal gait variability as the CV of stride time, i.e., stride time variability. We focused on stride variability as increased stride-to-stride fluctuations have been consistently linked with higher fall risk in older adults and neurological populations (42–46). The variability of spatial and temporal gait parameters for each trial was quantified using the following formula:

$$\begin{array}{l} \text{Coefficient of variation }\left(\text{\%}\right)=\left(\frac{\text{standard deviation}}{\text{mean}}\right)\times \;100. \end{array}$$

### Gait Speed

We computed the over-ground walking speed with the average speed across three trials.

### Ankle Movement Control

The ankle movement control involved tracking a sinusoidal target trajectory with isolated, ankle plantarflexion–dorsiflexion movements. The participant sat in an upright chair in front of a 32-inch monitor placed about 1.5 m away. The legs were positioned with hips and knees at ~90 degrees and ankle in a neutral position with 90 degrees between the fifth metatarsal and the fibula. Each foot rested on and was secured to the custom-built foot device with straps such that the axis of rotation of the ankle aligned with the axis of rotation of the device, ensuring simultaneous movement of the foot and the device.

### Task

The target ankle movement ranged from 15 degrees of dorsiflexion to 5 degrees of plantarflexion. The target trajectory of 0.3 Hz was displayed with a red line on the computer screen in front of the participants. Participants were asked to perform rhythmic ankle dorsiflexion and plantarflexion movements to match the target trajectory as accurately as possible. Real-time feedback of participants' ankle movement was displayed with a blue line. Each trial lasted for 35 s. Three familiarization trials preceded the test trials. Participants performed five consecutive test trials with each ankle. A rest period of 30 s was provided between test trials to minimize fatigue. Only the test trials were included in the data analyses. The task order for paretic and non-paretic legs was randomized across participants.

### Ankle Position Analyses

The ankle position was measured with a low-friction potentiometer (SP22G-5K, Mouser Electronics, Mansfield, TX, USA) with a sampling rate of 1,000 Hz (NI-DAQ card, Model USB6210, National Instruments, Austin, TX, USA), connected to the axis of rotation of the foot device. A custom-written algorithm in MATLAB (The Math Works Inc., Natick, MA, USA) controlled the visual presentation of each trial and computed the outcome variables.

### Ankle Motor Accuracy and Steadiness

We quantified the accuracy and steadiness of ankle movements on visuomotor tracking task by averaging across five test trials. The initial 10 s and the last 5 s of data from each trial were eliminated to allow for adjustment to the task and early cessation of the performance in anticipation of trial termination. The motor accuracy was measured using root mean squared error (RMSE) that quantified the distance between the target and the participant's ankle position trajectories. The motor steadiness was measured as the standard deviation (SD) of the participant's ankle position within each trial. For this, we first band-stop-filtered the position signal between 0.2 and 0.4 Hz to remove the task-related frequency of the 0.3-Hz sinusoidal target. Then, we quantified movement steadiness with the SD of the detrended position signal.

### Ankle Strength

We examined the ankle plantarflexion and dorsiflexion strength by measuring the isometric MVC force. Participants sat in an upright chair with the hip and knees at ~90 degrees and ankle in a neutral position. The maximum voluntary force was measured using a force transducer (Model 41BN, Honeywell, Morristown, NJ, USA) located parallel to the force direction on a customized foot device. Participants were instructed to exert maximum force at the ankle joint during plantarflexion or dorsiflexion for a period of 3 s while avoiding any extraneous movements at the knee, hip, and trunk. Three to five trials were performed for plantarflexion and dorsiflexion with the paretic and non-paretic legs. The MVC task order for leg condition (paretic and non-paretic legs) and contraction type (plantarflexion and dorsiflexion) was randomized across the participants. The strength was quantified as the maximum force generated across three to five trials.

### Statistical Analysis

All participants completed the training. To test the normality of pre and post-test outcome measures, we performed the Shapiro–Wilk test separately in both the training groups. To examine the influence of force-control training and strength training on gait outcomes, we used the generalized estimating equation (GEE) model for (1) stride length variability of the paretic leg, (2) stride length variability of the non-paretic leg, (3) mean stride length variability of both legs, (4) mean stride time variability of both legs, and (5) gait speed. Here, Training type (force-control and strength training) was the between-subject factor, and Time (pre-test and post-test) was the within-subject factor. To determine the task-specific effects of training, we used the GEE model on (1) the RMSE of ankle movement, (2) the SD of ankle movement, and (3) the MVC of plantarflexion and dorsiflexion for paretic and non-paretic legs. For the GEE models, we selected the unstructured working correlation matrix and identity or log link function based on the distribution of the data. Significant interactions were followed up with pairwise comparisons with Bonferroni corrections to assess the effect of trainings on gait outcomes, ankle motor accuracy, steadiness, and strength across the two groups. All analyses were performed using SPSS (SPSS Inc. version 24.0). The significance level was set at *p* < 0.05.

## Results

### Clinical Characteristics

The clinical characteristics of participants for each training group are displayed in Table 1. The independent *t*-test confirmed that the two groups had no significant difference in age (|*t*_20_*|* = 0.07, *p* = 0.94), time since stroke (|*t*_20_*|* = 0.90, *p* = 0.81), motor impairment level assessed through FMA-LE (|*t*_20_*|* = −1.05, *p* = 0.30), and cognitive status assessed using MoCA (|*t*_20_*|* = 0.65, *p* = 0.51).

**Table 1:** Demographics of the participants in each training group (mean ± SD).

	**Force-control training**	**Strength training**
	**(*N* = 11)**	**(*N* = 11)**
Age (years)	65.12 ± 11.08	64.72 ± 14.35
Sex (Females), *N*	4	4
Hemiparetic side (Right), *N*	8	10
Time since stroke (years)	7.36 ± 4.46	5.03 ± 5.76
**Lesion location**		
Cortical	8	6
Subcortical	2	2
Unknown	1	3
Walking aid, *N*	1	1
Orthosis, *N*	0	0
MoCA	24.00 ± 4.73	22.77 ± 4.36
FMA-LE	22.90 ± 9.15	26.46 ± 4.60

*MoCA, Montreal cognitive assessment (maximum score 30); FMA-LE, Fugl–Meyer motor assessment for lower extremity (maximum score 34); n/a, Not applicable. All scores are mean ± standard deviation*.

### Effects of Training Protocols on Stride Length Variability

**Figure 2:**
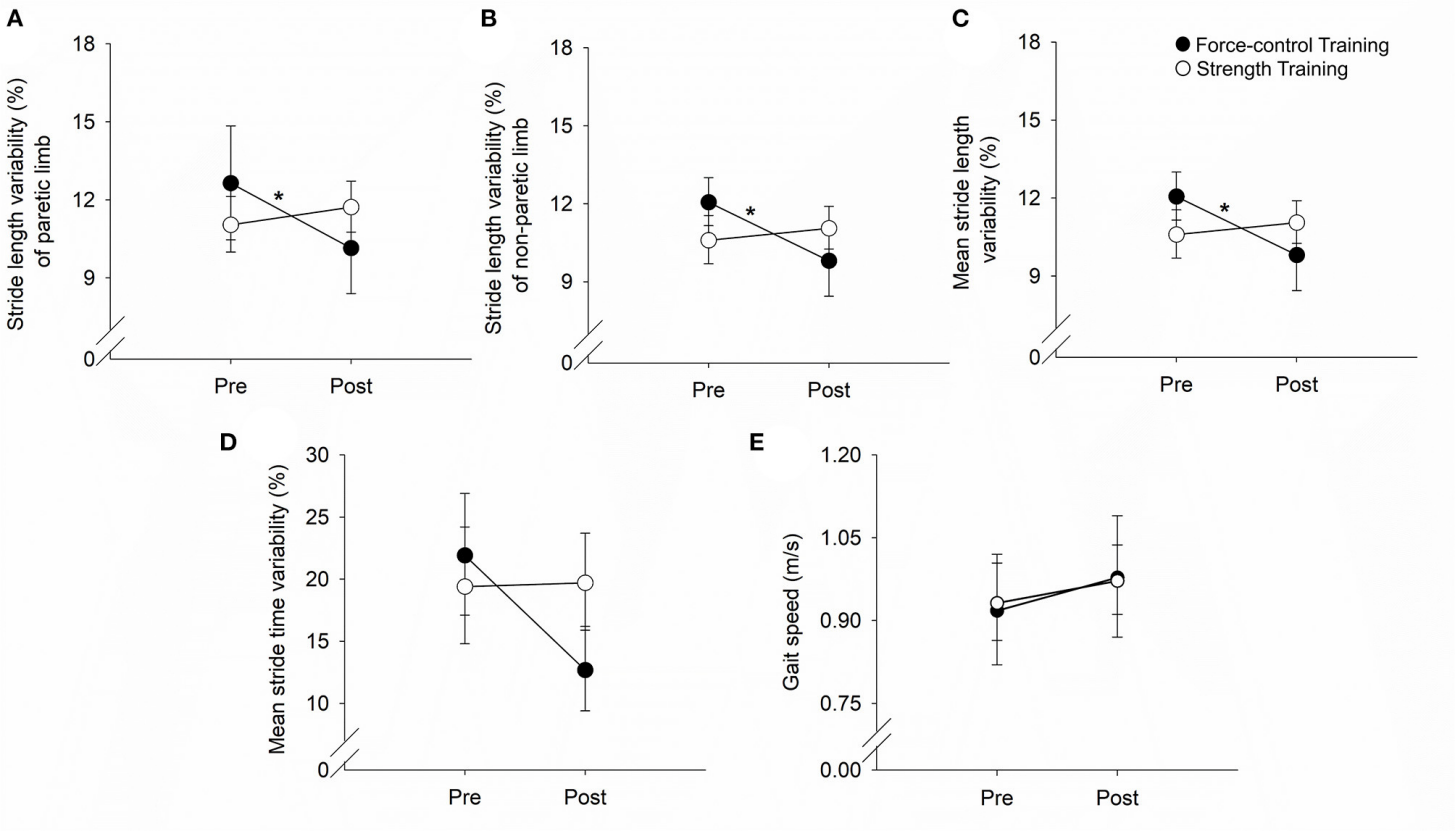
Effect of force-control and strength training protocols on stride length and stride time variability, and gait speed. Spatial gait variability was quantified as the coefficient of variation of the stride length of the paretic limb **(A)**, the stride length of the non-paretic limb **(B)**, and the mean stride length of the two limbs **(C)**. Temporal gait variability was quantified as the coefficient of variation of the mean stride time of the two limbs **(D)**. Spatial **(A–C)** gait variability reduced in the force-control training group but not in the strength training group. The temporal gait variability **(D)** trended (interaction, *p* = 0.05) to decline in the force-control training group but did not change in the strength training group. Gait speed **(E)** increased in both training groups. The figure shows significant interactions with *p < 0.05. Significant main effects are reported in the text.

Figure 2 demonstrates the results for stride length variability. For stride length variability of the paretic leg (Figure 2A), we found a significant time x training type interaction (Wald χ^2^ = 4.05, *p* = 0.04). *Post-hoc* comparisons of the two groups at the post-training session revealed no significant difference in stride length variability (*p* = 0.44). Importantly, however, the *post-hoc* comparisons on the training type revealed that stride length variability of the paretic leg reduced significantly after force-control training (*p* = 0.04). However, there was no change in stride length variability after strength training (*p* = 0.56). For stride length variability of the non-paretic leg (Figure 2B), we found a significant time × training type interaction (Wald χ^2^ = 3.98, *p* = 0.04). *Post-hoc* comparisons of the two groups at the post-training session revealed no significant differences in stride length variability of the non-paretic leg (*p* = 0.44). Notably, however, *post-hoc* analysis on the training type revealed that stride length variability of the non-paretic leg showed significant reduction following force-control training (*p* = 0.03) but not following strength training (*p* = 0.65). For mean stride length variability of both legs together (Figure 2C), we found a significant time x training type interaction (Wald χ^2^ = 4.13, *p* = 0.03). *Post-hoc* analysis showed that the mean stride length variability reduced significantly after force-control training (*p* = 0.02) but did not change after strength training (*p* = 0.70). At the post-training session, the two groups were not significantly different in mean stride length variability (*p* = 0.39).

### Effects of Training Protocols on Stride Time Variability

We found a close to significant time × training interaction (Wald χ^2^ =3.64, *p* = 0.056) for the stride time variability (Figure 2D). *Post-hoc* comparisons of the two groups at the post-training session showed no significant difference in stride time variability (*p* = 0.18). However, it is noteworthy that *post-hoc* comparisons on the training type showed that mean stride time variability reduced after force-control training (*p* = 0.03), but mean stride time variability did not change after strength training (*p* = 0.93).

### Effect of Training Protocols on Gait Speed

Figure 2E shows the effect of training protocols on gait speed. We found a significant main effect of time for gait speed (Wald χ^2^ = 8.06, *p* = 0.00), confirming that gait speed increased after both strength and force-control training. The time × training interaction on gait speed was not significant (Wald χ^2^ = 0.18, *p* = 0.66).

### Task-Specific Effects of Training Protocols on Ankle Motor Accuracy and Steadiness

**Figure 3:**
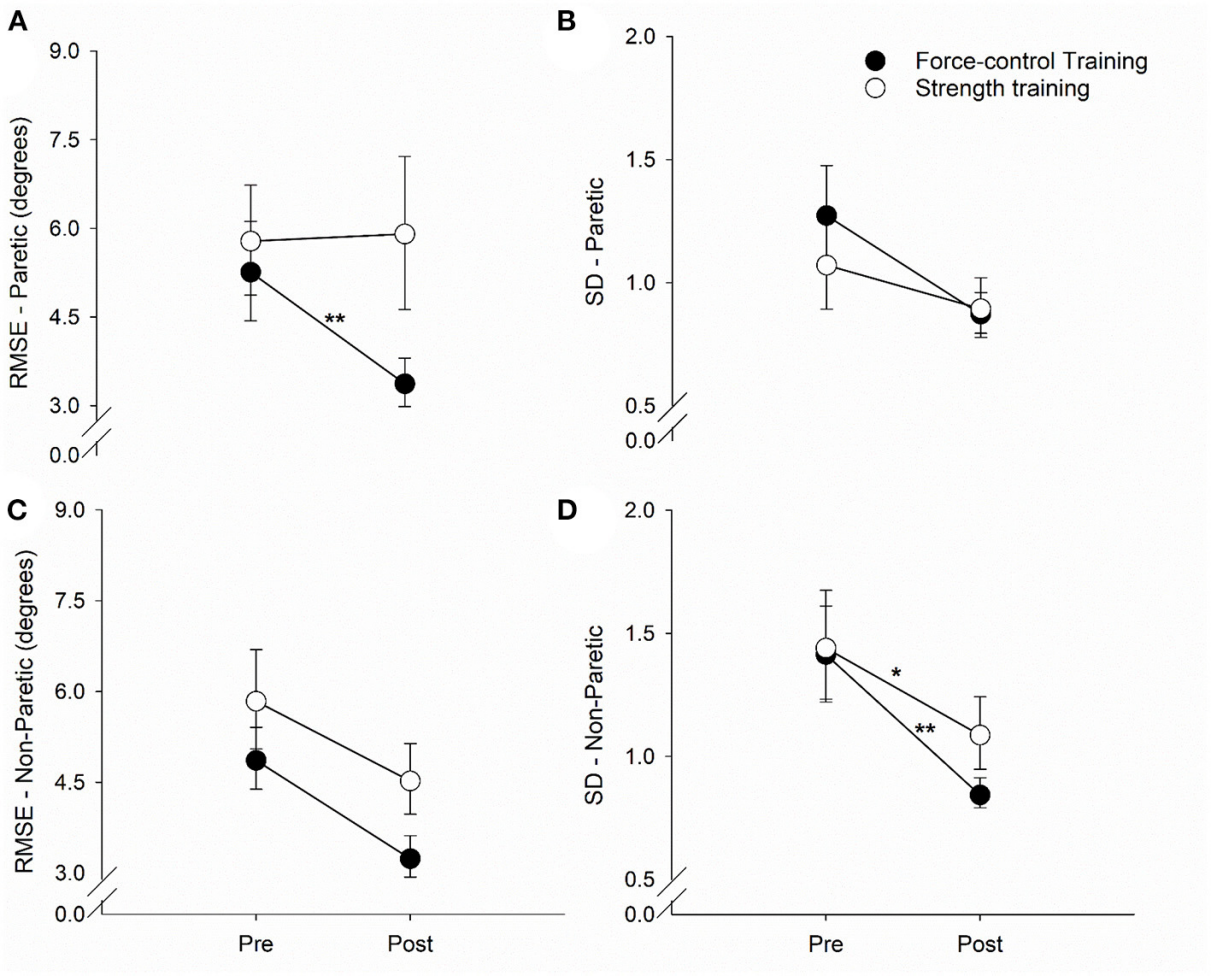
Effect of force-control and strength training protocols on ankle motor control. We quantified accuracy (root mean squared error, RMSE) and steadiness (standard deviation, SD) of plantarflexion and dorsiflexion. The force-control training group showed a decrease in the ankle RMSE of the paretic leg **(A)**. Overall, both training groups showed a reduction in the ankle RMSE of the non-paretic leg **(C)** and the SD of the paretic **(B)** and non-paretic leg **(D)**. Nonetheless, there was greater reduction in the SD of the non-paretic leg in the force-control group **(D)**. The figure shows significant interactions with *p < 0.05 and **p < 0.01. Significant main effects are reported in the text.

Figure 3 demonstrates the results for motor accuracy and variability for the paretic and non-paretic legs.

### Motor Accuracy

For the accuracy of the paretic ankle (Figure 3A), we found a significant main effect of time (Wald χ^2^ = 10.02, *p* = 0.00) suggesting that the RMSE reduced post-training. We also found a significant time x training type interaction (Wald χ^2^ = 13.83, *p* = 0.00) on the accuracy of the paretic ankle. *Post-hoc* analysis showed that the RMSE of the paretic ankle significantly reduced after force-control training (*p* = 0.00) but not after strength training (*p* = 0.83). For the accuracy of the non-paretic ankle (Figure 3C), we found a significant main effect of time (Wald χ^2^ = 21.94, *p* = 0.00), suggesting that the RMSE of the non-paretic leg reduced after both the trainings.

### Motor Steadiness

For the steadiness of the paretic ankle (Figure 3B), we found a significant main effect of time (Wald χ^2^ = 5.41, *p* = 0.02), suggesting that the SD of the paretic ankle reduced after training. For the steadiness of the non-paretic ankle (Figure 3D), we found a significant main effect of time (Wald χ^2^ = 46.12, *p* = 0.00), suggesting that the SD of the non-paretic ankle reduced after training. We also found a significant time x training type interaction (Wald χ^2^ = 3.98, *p* = 0.04) on the steadiness of the non-paretic ankle. *Post-hoc* analysis revealed that non-paretic SD showed a decline after both force-control training (*p* = 0.00) and strength training (*p* = 0.01), with greater decrease evident in the force-control training group (Figure 3D).

### Task-Specific Effects of Trainings on Ankle Strength

**Figure 4:**
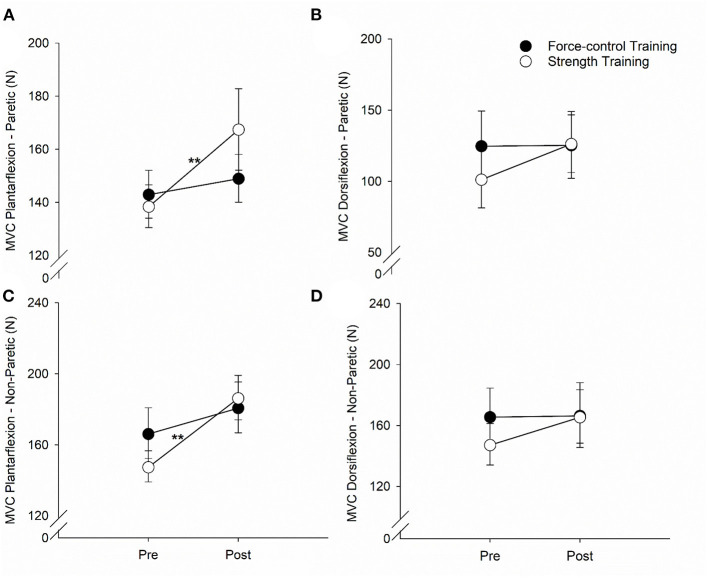
Effect of force-control and strength training protocols on ankle strength. We quantified plantarflexion and dorsiflexion strength as the force during maximum voluntary contractions (MVC). The strength training group showed increase in plantarflexion MVC of the paretic limb **(A)**, dorsiflexion MVC of the paretic limb **(B)**, and plantarflexion MVC of the non-paretic limb **(C)**. There was no change in the dorsiflexion MVC of the non-paretic limb with training **(D)**. The figure shows significant interactions with **p < 0.01. Significant main effects are reported in the text.

Figure 4 demonstrates the results for ankle plantarflexion and dorsiflexion strength for paretic and non-paretic legs. For the plantarflexion strength of the paretic leg (Figure 4A), we found a significant main effect of time (Wald χ^2^ = 12.81, *p* = 0.00), suggesting that the overall plantarflexion MVC of the paretic leg increased after training. We also found a significant time x training type interaction on the plantarflexion strength (Wald χ^2^ = 5.81, *p* = 0.01**)**. *Post-hoc* comparisons showed that the plantarflexion MVC of the paretic leg increased significantly after strength training (*p* = 0.00) but remained unchanged after force-control training (*p* = 0.43). For the dorsiflexion strength of the paretic leg (Figure 4B), there was a trend toward time x training type interaction (Wald χ^2^ = 3.30, *p* = 0.069). The dorsiflexion MVC of the paretic leg appeared to increase after strength training (*p* = 0.01) but remained unchanged after force-control training (*p* = 0.94).

For the plantarflexion strength of the non-paretic leg (Figure 4C), we found a significant main effect of time (Wald χ^2^ = 24.08, *p* = 0.00), suggesting that the plantarflexion MVC of the non-paretic leg increased after training. We found a significant time × training type interaction (Wald χ^2^ = 5.00, *p* = 0.02) on the plantarflexion strength of the non-paretic leg. *Post-hoc* comparisons revealed that the plantarflexion MVC of the non-paretic leg showed significant increase after strength training (*p* = 0.00), but not after force-control training (*p* = 0.08). The dorsiflexion strength of the non-paretic leg (Figure 4D) did not show any significant main effects (Time: Wald χ^2^ = 2.74, *p* = 0.09; Training type: Wald χ^2^ = 0.16, *p* = 0.68) or interaction (Wald χ^2^ = 2.29, *p* = 0.13). The mean values for all the gait variables at pre- and post-training sessions for the two groups are shown in Supplementary Table S1.

## Discussion

The purpose of this study was to compare the effect of force-control training and strength training on the spatiotemporal measures of gait variability as well as gait speed among ambulatory chronic stroke survivors. We found that stride length and stride time variability were significantly reduced after force-control training, but not after strength training. Both the training protocols improved gait speed in stroke survivors. The current study is the first investigation in chronic stroke survivors that compares the effectiveness of two distinct motor interventions to improve gait variability. Although both force-control and strength training protocols improved gait speed, improvements in gait variability were evident with force-control training, but not with strength training. Thus, we provide novel evidence that force-control training may offer a potential approach in reducing gait variability and promoting safe mobility in individuals with chronic stroke.

### Force-Control vs. Strength Training for Gait Variability

The unsteady gait patterns following stroke may reflect reduced ability to adapt walking to ongoing internal and external perturbations and contribute to poor balance (47, 48). Interventions that re-train strategies to reduce gait variability are necessary to promote functional independence in stroke survivors. A key finding of our study is that stride length and stride time variability significantly reduced after force-control training but remained unchanged after strength training. The majority of the individuals in the force-control training group (90.90%) showed a decline in stride variability compared with the strength training group (54.54%) (Supplementary Figure S1). The next few paragraphs delineate the key differences in force-control and strength training protocols that may explain the differential effects of these trainings on stride variability.

The force-control training focused on improving the accuracy and consistency of submaximal ankle forces ranging from 5 to 25% of MVC. In contrast, strength training targeted increase in the maximal force generation capacity of the ankle muscles by training the participants to produce forces ranging from 65 to 80% of MVC. Furthermore, force-control training involved rhythmic force modulation through gradual increase and decrease of ankle forces to match a dynamic target force, whereas strength training focused on ballistic muscle contractions for a short duration. The task-specific effects of the training (Figure 3) suggest that force-control training, but not strength training, enhanced the ability to precisely modulate ankle dorsiflexion and plantarflexion forces, the key muscles required to maintain a steady walking pattern (17, 18, 49). Such decreased error in movement execution through force-control training potentially contributed to a less variable gait pattern (5).

Second, force-control training involved modulating submaximal forces at varying rates by changing the frequency of the sinusoidal target in successive sessions. Practicing modulation of submaximal forces at varying rates emulates rhythmic ankle dorsiflexion and plantarflexion forces involved in walking at different speeds and terrains. In strength training, the level of the target was increased in consecutive sessions for training participants to produce a greater magnitude of ankle forces. While the generation of sufficient muscle strength is important for increasing walking speed (50–52), strength training does not necessarily translate into the steady modulation of temporal and spatial stride parameters. These findings are in line with our previous work that indicated no relationship between strength and gait variability in high-functioning stroke survivors (15).

Third, force-control training emphasized on constant visuomotor integration such that muscle contractions were scaled in response to the visual target. In contrast, the strength training group received brief visual feedback about reaching the target. Possibly, force-control tasks implicitly trained visuomotor integration that constitutes an important aspect of adapting a walking pattern in different environments. Taken together, our data suggest that an intervention focusing on increasing force generation capacity such as that involved in strength training may not be sufficient for improving the controlled modulation of ankle forces required for walking, thus emphasizing the utility of force-control training in reducing gait variability in chronic stroke survivors.

### Both Motor Training Protocols Improve Gait Speed

Our findings suggest that both force-control and strength training groups showed a modest but significant improvement in gait speed. Locomotor recovery after stroke is frequently quantified with gains in walking speed and walking distance (53, 54). However, despite the substantial recovery of walking speed, impairments in gait variability may persist (48). Thus, gait speed and gait variability represent different constructs of walking. While gait velocity may be an important outcome to determine walking capacity, the importance of gait variability in representing walking stability and safe mobility cannot be undermined. In the current study, while both trainings increased walking speed, only the force-control training positively influenced gait variability. These findings provide initial evidence that improvements in gait variability may not be obtained as a spin-off effect of a locomotor intervention designed to target gait speed but may require specific considerations.

### Task-Specific Changes in Motor Function

In both training groups, we found task-specific changes in motor function suggesting that the training groups improved specifically on the task they were trained for. The force-control training group demonstrated enhanced ankle motor control indicated by improved accuracy (reduced RMSE) and steadiness (reduced SD) of plantarflexion and dorsiflexion movements of the paretic and non-paretic legs. Only a few studies have investigated interventions for improving accuracy and steadiness of lower limb movements after stroke (49, 55, 56); however, none of these studies investigated the functional consequence of such intervention on gait variability. Previously, we showed that reduced accuracy of single-joint goal-directed ankle movements after stroke is related to the multi-joint walking function in stroke survivors (27). Current findings highlight that a training focused on ankle force modulation facilitates improvement in the accuracy and consistency of voluntary ankle control during isolated, singe-joint movements as well as multi-joint movements involved in walking. The strength training group showed a significant increase in ankle plantarflexion strength of the paretic and non-paretic legs, and dorsiflexion strength of the paretic leg. In contrast to the strength training group (overall 24.24% change in MVC), the force-control training group (overall 7.60% change in MVC) showed minimal, non-significant change in ankle strength, suggesting that strength gains were limited to the strength training group. These findings concur with previous studies showing immediate improvements in ankle strength in the both limbs after isokinetic or isometric progressive resistance training (32, 57).

Finally, we found some cross-training effects of strength training on general improvements in ankle movement accuracy and steadiness, even though force-control training did not facilitate any significant changes in ankle strength. These results are in line with previous studies that showed that higher strength contributes to a more steady motor output (36, 58). Nevertheless, the magnitude of improvement in movement accuracy and steadiness was greater in force-control training (average 34.4% change in accuracy and 35.7% change in steadiness across both limbs) as compared with strength training (average 10.2% change in ankle accuracy and 20.4% change in ankle steadiness across both limbs). Thus, force-control training was superior to strength training in improving ankle accuracy and steadiness.

### Considerations

This proof-of-concept study in a relatively small sample of stroke survivors was not sufficiently powered to detect changes in all the measures of gait variability. This preliminary study provides initial evidence for small but statistically significant improvements in stride length variability with relatively short duration of force-control training. Due to lack of research on interventions to improve gait variability after stroke, our study was designed as a proof-of-concept study to test whether force-control or strength training of ankle joint can improve stride-to-stride variability. This work was based on our previous findings showing that the motor accuracy of the single joint (i.e., ankles) in stroke survivors is associated with over-ground walking, a multi-joint function (15, 27). Whether such changes in stride variability are clinically meaningful and the impact of training on variability in other gait parameters remain unclear. We did not find any significant correlations between the change in gait variability and change in force control or strength outcomes following training (see Supplementary Figure S2). The current study is likely underpowered to effectively elucidate the relationship between changes in stride length variability and ankle outcome measures. Thus, randomized controlled trials in larger populations will be required to corroborate our findings. Furthermore, with only one participant in each group using a walking aid, we were unable to determine the effect of walking aid on training-related changes in stride variability. The study cohort had relatively mild to moderate degree of motor impairment as evident from the FMA-LE score. Future studies should investigate the effectiveness of these interventions for gait variability in more severely impaired chronic stroke survivors.

Compared with the typical training duration (30–54 h) applied in locomotor training protocols (9, 34, 37), the current study involved relatively short training duration (6 h). Perhaps, the improvements in gait variability were relatively modest because of the limited duration of training. The training duration per session was relatively longer (~90 min) than typical therapy sessions (~45 min) in the clinics (59). Future studies investigating the dose-response effect for force-control training on gait variability in stroke is warranted. While our study shows immediate improvement in gait variability with a relatively brief training period, we did not investigate the retention of training effects with follow-up sessions. Future study designs with longer follow-up period are needed to inform the training dose that can facilitate long-term retention of improvements in gait variability. Furthermore, the neuromuscular mechanisms underlying force-control training that could contribute to reduced gait variability should be investigated to enhance the effectiveness of such interventions. Finally, the functional consequence of improved gait variability on fall risk is an important area of future investigation in stroke motor recovery.

## Conclusion

Our study provides novel evidence that force-control training is more effective than strength training in reducing gait variability in chronic stroke survivors. Force-control training resulted in improved motor accuracy and steadiness that translated into reduction in spatial and temporal gait variability. Gait variability remained unaltered following strength training, although both training protocols led to similar improvements in gait speed. Increased gait variability is highly predictive of poor balance and heightened risk of falls (42, 45, 60, 61). Interventions focused on improving force modulation rather than increasing force generation capacity may offer a promising rehabilitation approach for reducing gait variability and promoting safe mobility after in chronic stroke survivors.

## Data Availability Statement

The raw data supporting the conclusions of this article will be made available by the authors, without undue reservation.

## Ethics Statement

The studies involving human participants were reviewed and approved by Institutional Review Board of the University of Florida. The patients/participants provided their written informed consent to participate in this study.

## Author Contributions

NL and EC conceived and designed the experiments. NL and AC-M conducted data collection. PP conducted data analysis. PP and NL did data interpretation and drafted the manuscript. PP, AC-M, EC, and NL revised and approved the manuscript for publication. All authors contributed to the article and approved the submitted version.

## Conflict of Interest

The authors declare that the research was conducted in the absence of any commercial or financial relationships that could be construed as a potential conflict of interest.
