# Supplementary material for: Force-Control vs. Strength Training: The Effect on Gait Variability in Stroke Survivors
Source: Front Neurol. 2021 Jul 15;12:667340. doi: 10.3389/fneur.2021.667340 (PMC8319601; doi:10.3389/fneur.2021.667340)
Supplement: Supplementary file 1 [file Data_Sheet_1.pdf]

Table S1 Mean (SE) for gait outcome measures at pre- and post-training sessions for the force-control and strength training groups.

Variable	Force-control training (N=11)		Strength training (N=11)		p_{time}	p_{group}	$p_{time\ x\ group}$
	Pre	Post	Pre	Post			
Stride length CV % (paretic leg)	12.66±2.19	10.17±1.77	11.06±1.07	11.74±0.98	0.249	0.170	**0.044**
Stride length CV % (non-paretic leg)	12.08±0.92	9.83±1.39	10.62±0.93	11.08±0.82	0.187	0.052	**0.046**
Stride length CV % (mean of two legs)	11.70±1.48	9.61±1.40	10.67±0.93	11.00±0.78	0.132	0.156	**0.038**
Stride time CV % (mean of two legs)	22.00±4.90	12.80±3.40	19.50±4.70	19.80±3.90	0.073	0.562	0.056
Gait speed (m/s)	0.92±0.10	0.98±0.11	0.93±0.07	0.97±0.06	**0.005**	0.976	0.668
RMSE (degrees) (paretic leg)	5.27±0.84	3.39±0.41	5.80±0.93	5.92±0.1.29	**0.002**	0.096	**0.000**
RMSE (degrees) (non-paretic leg)	4.89±0.51	3.27±0.32	5.87±0.82	4.55±0.58	**0.000**	0.626	0.123
SD (paretic leg)	1.27±0.19	0.87±0.08	1.07±0.18	0.89±0.12	**0.020**	0.897	0.218
SD (non-paretic leg)	1.42±0.18	0.85±0.06	1.44±0.22	1.09±0.14	**0.000**	0.409	**0.046**
MVC Plantarflexion(N) (paretic leg)	143.02±9.01	149.03±9.00	138.51±8.06	167.47±15.34	**0.000**	0.485	**0.016**
MVC Plantarflexion(N) (non-paretic leg)	166.59±14.24	181.08±14.31	147.24±8.81	186.63±12.51	**0.000**	0.683	**0.025**
MVC Dorsiflexion(N) (paretic leg)	124.87±24.50	125.52±23.52	101.37±20.07	126.41±20.26	0.056	0.885	0.069
MVC Dorsiflexion(N) (non-paretic leg)	166.03±18.53	166.84±21.32	147.69±13.67	165.92±17.49	0.098	0.683	0.130

CV, coefficient of variation; RMSE, root mean squared error; SD, standard deviation; MVC, maximum voluntary contraction force; N, Newton.
p indicates the statistical significance value for main effects and interactions.

Figure S1. Change in mean stride length variability and mean stride time variability for individual participants in each group.

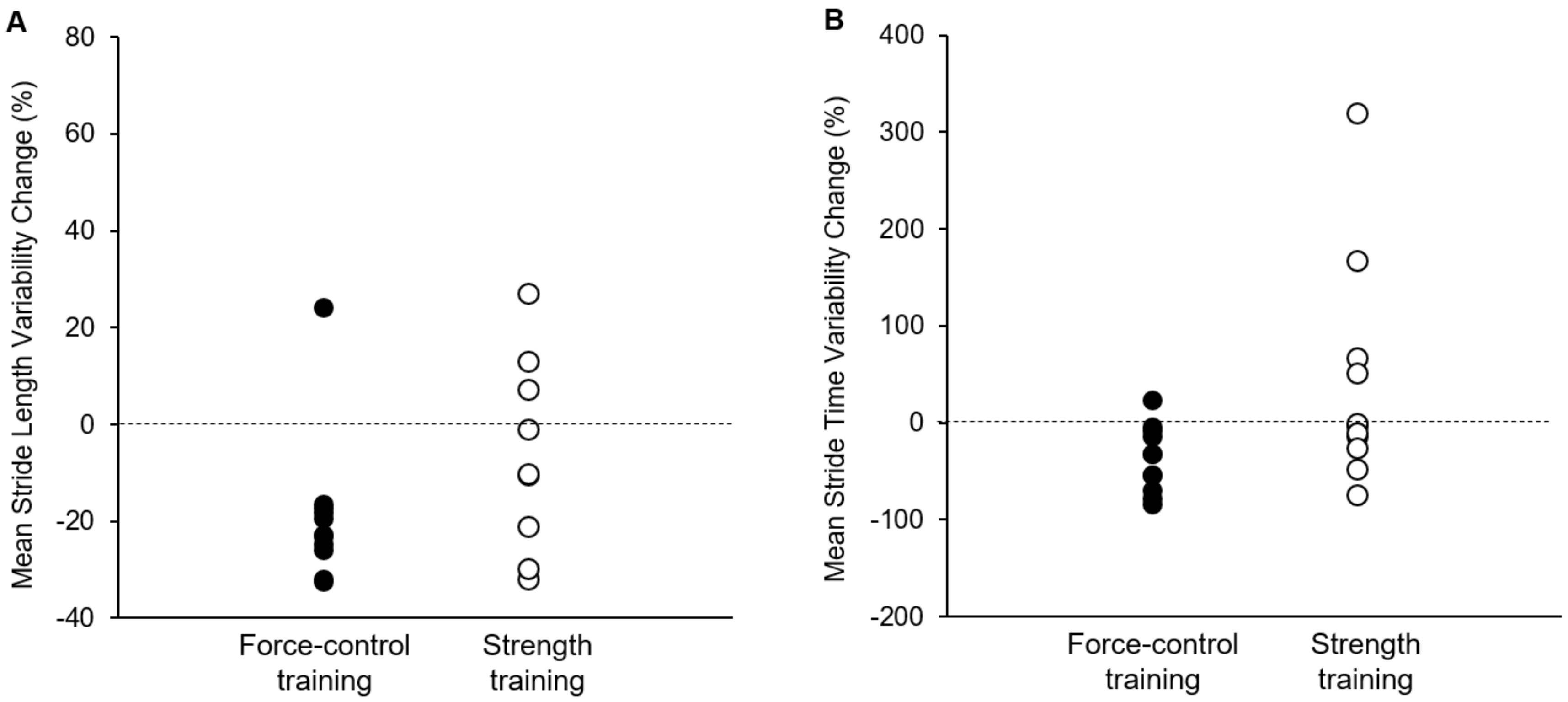

The Pre- to Post-training change score was computed using the formula (Post – Pre)/Pre x 100. Negative change scores for (A) mean stride length variability and (B) mean stride time variability indicates improved performance. The force-control training group showed decline in stride length variability and stride time variability more consistently and to higher magnitude than the strength training group.

Figure S2. Relationship between change in mean stride length variability and change in force-control and strength outcomes for the training groups.

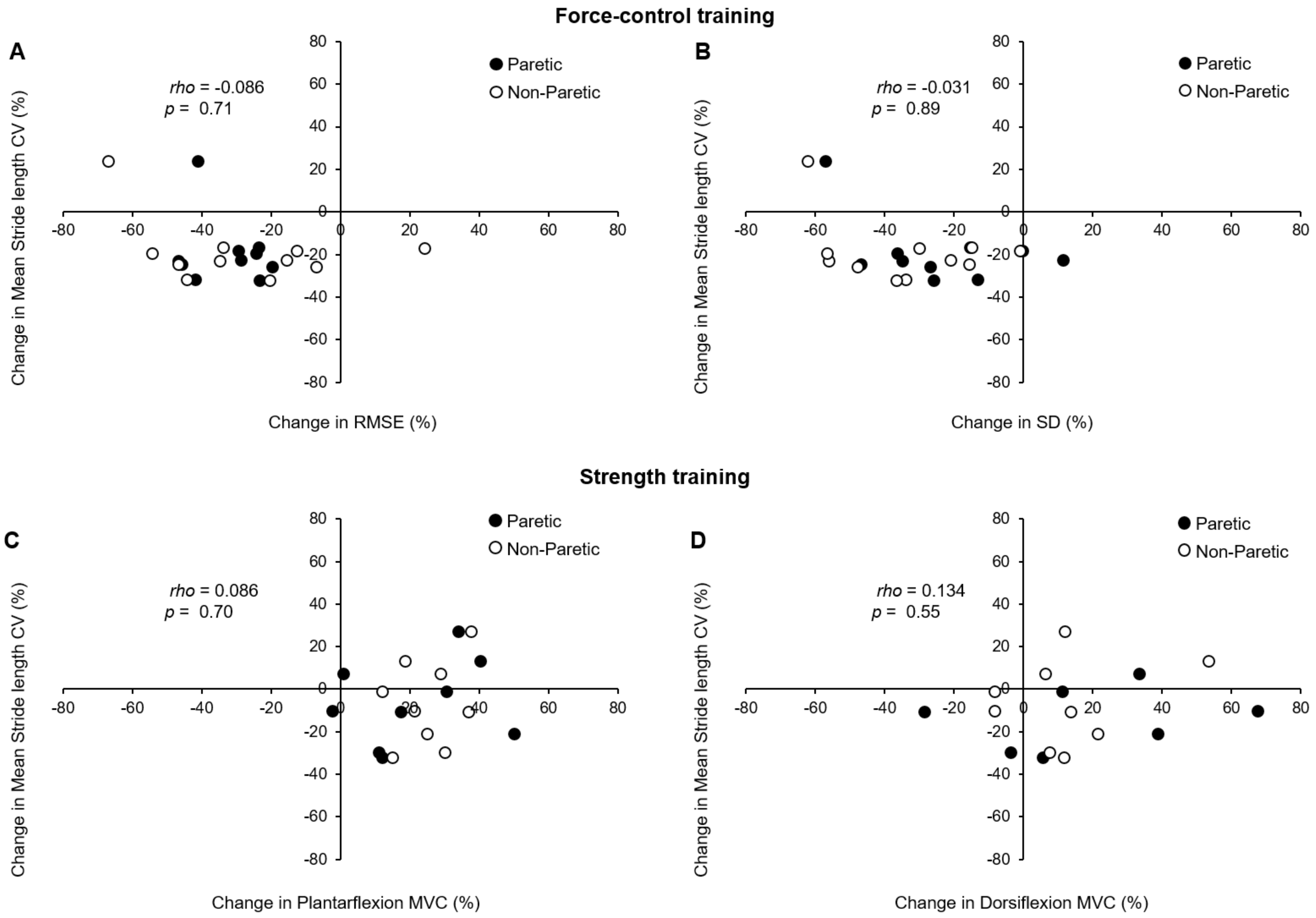

Spearman's correlation between pre- to post-training change in mean stride length variability versus (A) change in RMSE and (B) change in SD in force-control training group. Spearman's correlation between pre- to post-training change in mean stride length variability versus (C) change in ankle plantarflexion MVC and (D) change in dorsiflexion MVC in strength training group. The change score was computed using the formula (Post – Pre)/Pre x 100. We did not find any significant correlations between the change in gait variability and change in force control or strength outcomes following training.
